# Heat stress related dairy cow mortality during heat waves and control periods in rural Southern Ontario from 2010–2012

**DOI:** 10.1186/s12917-015-0607-2

**Published:** 2015-11-27

**Authors:** Katherine E. Bishop-Williams, Olaf Berke, David L. Pearl, Karen Hand, David F. Kelton

**Affiliations:** Department of Population Medicine, Ontario Veterinary College, University of Guelph, 50 Stone Rd East, Guelph, ON N1G 2 W1 Canada; Department of Mathematics and Statistics, University of Guelph, Univof Guelph, 50 Stone Rd East, Guelph, ON N1G 2 W1 Canada; Institute of Biometry, Epidemiology and Information Processing, University of Veterinary Medicine, Hanover, Germany; Strategic Solutions Group, 142 Hume Rd, Puslinch, ON Canada

**Keywords:** Southern Ontario, Heat stress, Rural, Dairy cow, Mortality

## Abstract

**Background:**

Heat stress is a physiological response to extreme environmental heat such as heat waves. Heat stress can result in mortality in dairy cows when extreme heat is both rapidly changing and has a long duration. As a result of climate change, heat waves, which are defined as 3 days of temperatures of 32 °C or above, are an increasingly frequent extreme weather phenomenon in Southern Ontario. Heat waves are increasing the risk for on-farm dairy cow mortality in Southern Ontario. Heat stress indices (HSIs) are generally based on temperature and humidity and provide a relative measure of discomfort which can be used to predict increased risk of on-farm dairy cow mortality. In what follows, the heat stress distribution was described over space and presented with maps. Similarly, on-farm mortality was described and mapped. The goal of this study was to demonstrate that heat waves and related HSI increases during 2010–2012 were associated with increased on-farm dairy cow mortality in Southern Ontario.

Mortality records and farm locations for all farms registered in the CanWest Dairy Herd Improvement Program in Southern Ontario were retrieved for 3 heat waves and 6 three-day control periods from 2010 to 2012. A random sample of controls (2:1) was taken from the data set to create a risk-based hybrid design. On-farm heat stress was estimated using data from 37 weather stations and subsequently interpolated across Southern Ontario by geostatistical kriging. A Poisson regression model was applied to assess the on-farm mortality in relation to varying levels of the HSI.

**Results:**

For every one unit increase in HSI the on-farm mortality rate across Southern Ontario increases by 1.03 times (CI_95%_ (IRR) = (1.025,1.035); *p* = ≤ 0.001). With a typical 8.6 unit increase in HSI from a control period to a heat wave, mortality rates are predicted to increase by 1.27 times.

**Conclusions:**

Southern Ontario was affected by heat waves, as demonstrated by high levels of heat stress and increased on-farm mortality. Farmers should be aware of these risks, and informed of appropriate methods to mitigate such risks.

## Background

Heat stress is defined as a physiological response to environmental heat which can cause such signs or symptoms as discomfort, increased respiration rate, dehydration, changes in cardiac function, and in severe cases may result in death [1]. Heat stress occurs not only in humans, but in livestock species as well. Documented heat stress related mortality has occurred in sows [2], chickens [3] and in dairy cattle [4].

Dairy cows are highly susceptible to changes in temperature, particularly to rapid increases in temperature or long periods of extreme heat. Recent increases in production, which result in higher metabolic rates, have exacerbated this vulnerability to extreme heat in dairy cows [5]. This susceptibility can result in losses in productivity of 1.5 to 2 L of milk per cow per day [6], and increased morbidity and increased mortality [4]. For the average dairy cow farmer in Southern Ontario, a 3-day heat wave would result in losses of $253–$337 based on average milk prices of $0.75 /L and farm sizes of 75 cows in milking. For a farm where one cow dies as a result of the heat wave (excess death) the economic impact is between $2,000–$2,500 dollars. In the United States, the economic impacts related to heat stress in dairy cows have been estimated to be approximately $897 million in losses per year [7].

Heat related dairy cow mortality is not only a problem of the future. Rather, mortality caused by heat waves is a growing concern, dating back decades. Prior research in California demonstrated that during periods of high temperature in the summer, on-farm calf mortality increased substantially [8]. In Italy, a threshold for increased dairy cow mortality was established based on a combinatory estimator of heat and humidity related discomfort known as the heat stress index (HSI). Vitali et al. [4] determined that increased dairy cow mortality was expected in Southern Italy when the HSI increased above 70 units. In Hungary, models of future heat stress days predict increases of up to 27 heat stress days per year in areas with substantial cattle farming by the year 2040. This rise in heat stress days is expected to increase the on-farm mortality dramatically [9]. A study in the United States proposed that the impacts of heat waves on mortality are increasing as per cow production increases resulting in increases in the cow’s metabolic rate and metabolic heat production [5].

A heat wave is defined by Environment Canada as 3 consecutive days of temperatures of at least 32 °C daily maximum temperature [10]; however, a variety of other definitions exist for a heat wave. A heat wave by this definition combined with any level of humidity is sufficient to cause an HSI of at least 70 units based on the Dairy Cow Heat Stress Indicator developed by Johnson and Vanjonack [11]. The Dairy Cow HSI estimates heat stress using parameters known as the dry bulb temperature and the relative humidity readings [11]. As a result, a heat wave is commonly associated with heat stress in individuals, although not all cows will experience heat stress characteristics beyond the 70 unit threshold. Instead, heat stress is more common in individuals when the HSI is above the threshold, and can be more pronounced leading to increased risk of on-farm mortality [4].

The data for this study came from databases maintained by Dairy Farmers of Ontario [12] and CanWest Dairy Herd Improvement (DHI). All farms in Southern Ontario are licensed to produce and sell milk by the DFO, but participation in milk recording with DHI is voluntary. In Canada, CanWest DHI [13] offers value added services to dairy farmers such as pregnancy testing, disease testing and computerized record keeping. This pay for service registered program involved 2,898 of 4,084 (71 %) dairy farms in Ontario in 2009 [14]. Records kept by DHI greatly exceed industry requirements and provide a thorough description of what is occurring at the farm level.

The predictions regarding the future impacts of heat waves consistently point to increases in frequency, intensity and duration [15–18]. With increased severity of heat waves there is expected to be an increased impact of heat waves on health and increases in the economic impact these heat waves have in Southern Ontario and specifically on the dairy industry.

Poisson regression modeling can be used to estimate the incidence rate ratio (IRR) or mortality rate from counts of death records such as those collected by the DHI. Statistical modelling of spatially correlated data such as heat stress [19] can be used to better understand these count data.

The goal of this study was to understand the current impact that heat waves during 201–2012 have had on mortality rates of dairy cows on farms in Southern Ontario. Specifically, the objectives of this study were: (i) to collect and summarize mortality data for dairy farms in Southern Ontario during heat waves and control periods; (ii) to estimate the increase in mortality rate of dairy cows on-farm in Southern Ontario as the HSI increases; and (iii) to determine how risk for on-farm dairy cow mortality varied across Southern Ontario during heat waves.

## Methods

### Data collection

Heat waves were identified using a search of the popular press, which indicated possible heat wave dates. As a result of the variability in definitions for a heat wave, each heat wave was confirmed to meet the Environment Canada definition of a heat wave (simultaneously at 3 different weather stations) between 2010 and 2012. Of the 6 popular press heat waves identified, 4 (66 %) met the Environment Canada definition of a heat wave, including our extension of the definition over space, to meet the definition at 3 or more weather stations. An a priori decision was made to include only the first heat wave in case that more than one heat wave occurred in any given year, thus allowing for a maximum of 3 heat waves in 3 years. Due to the variability in definitions of heat waves, no comprehensive search could be conducted. Environment Canada does not report heat waves in its extreme weather database. Control periods were 3-day periods selected based on dates of confirmed heat waves to start 3 weeks (21 days) prior to the start of the heat wave and 3 weeks following the start of the heat wave (i.e. if the first day of the heat wave was a Monday, so too are the first days of the control periods). Two of the heat waves identified were longer than 3 days (4 days each). Only the first 3 days were accounted for to show consistency. Therefore, there were 3 heat waves of 3 days each, and 6 control periods of 3 days each (Table 1). As a result, the estimates are the minimum anticipated impact of a heat wave in Southern Ontario. Control periods were matched for day of the week to control potential biases for daily activities on farms. By matching control periods for day-of-the-week, it reduces the likelihood that particular weekly stressors confound the heat stress effect. Control periods were used to compare the excess mortality during heat waves as heat-related illnesses and deaths are not and cannot be recorded in the DHI databases.

**Table 1:** Dates of exposure (heat waves) and control periods for each of 2010-2012

Exposure period	Control period 1 (Preceding)	Control period 2 (Following)
June 19–21, 2012	May 29–31, 2012	July 10–12, 2012
July 20–22, 2011	June 29–July 1, 2011	Aug 17–19, 2011
July 5–7, 2010	June 14–16, 2010	Aug 2–4, 2010

Heat stress maps were created as part of a previous study to map the distribution of heat stress across Southern Ontario [19]. Briefly, weather data were obtained from the National Climate Data and Information Archive [10] for 37 hourly weather stations across Southern Ontario on 27 dates (i.e., 3 heat waves and six 3-day control periods) and the hourly HSI was estimated for each point location.

The HSI was estimated using the Dairy Cow HSI developed by Johnson and Vanjonack [11]:

$$ \mathrm{H}\mathrm{S}\mathrm{I} = {T}_{DB} + \left({T}_{DP}*\ 0.36\right) + 41.5 $$

where *T*_*DB*_ and *T*_*DP*_ denote dry bulb temperature and dew point temperature in degrees Celsius, respectively. In a study comparing 5 heat stress indicators across 105 cities, including the HSI, Barnett et al. [20] concluded that all indices are equally predictive of heat stress and its resultant impacts. They thus proposed to choose an index based on data availability. Therefore, in this study, the HSI as defined above, was chosen over the commonly used Temperature Humidity Index (THI).

The average daily maximum heat stress for each point location was estimated by averaging the true daily maximum HSI from each day over the 3-day period. Ordinary geostatistical kriging with exponential semivariogram structures was used to spatially predict the 3-day average over the maximum daily HSI during 3 heat waves and 6 control periods across Southern Ontario.

Individual dairy cow and farm data were collected from the CanWest DHI and the DFO databases in 2013. Data collected from the CanWest DHI database included mortality records, status upon leaving the farm, herd size, breed and birth date (age). Exact global positioning system (GPS) location data for each farm was collected from the DFO database, along with a herd identification number. Farms were matched by the DFO identification number, which is captured by the DHI database. Farm coordinates measured by GPS and mortality records were used to create a point map of on-farm deaths for each of the 3 years.

Farm locations were used in conjunction with heat stress maps to predict the level of heat stress at each farm location (i.e. the level of heat stress at each farm was set equal to the kriging predicted HSI).

A map of dairy cow farm density was created using DHI and DFO location data in ArcGIS version 10 [21], aggregated at the county level.

### Ethics and data availability

The Research Ethics Board at the University of Guelph did not require ethics approval for this study. Data acquired from the DHI and DFO databases was accessed through Strategic Solutions (Puslinch, ON, Canada).

### Modelling and analysis

**Fig. 1:**
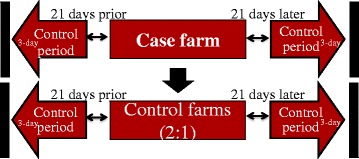
Farm selection protocol for observational hybrid study design examining changes in on-farm mortality rate in dairy cows in Southern Ontario from 2010–2012

All farms with a death event during a heat wave (case farms) were included in the study. Two random control farms were selected for each case farm, without matching, during the same heat wave period. Control farms were selected from the pool of dairy farms not reporting a death during the heat wave, which were registered with CanWest DHI during both control periods. Farms were selected using a random number generator and were chosen with replacement for each year (i.e. farms could be selected only once per year but selected again as a control for other years). The dataset of 2 controls to each mortality record were used to create a risk-based hybrid design where all case farms and control farms were compared during the prospective and retrospective control periods. The term risk-based hybrid design is intended to imply that all cases from an exposure were selected for inclusion, as well as a ratio of 2 control farms for each. This hybrid study design borrows from case–control design along the centre vertical line of the sampling frame depiction (Fig. 1), and from a case-crossover design in the horizontal lines of the depiction. A case–control study design would not have allowed for the estimation of mortality rates; however, the use of case and control farms created a cohort from which excess mortality rates during a heat wave as compared to the two control periods can be estimated. In this study, the excess mortality during (the first) heat waves in 2010–2012 was of interest. Refer to Fig. 1 for a detailed illustration of the sampling for this study.

Quasi-likelihood estimation was used in a Poisson modelling framework rather than maximum likelihood estimation to handle potential over-dispersion. A Poisson model assumes that the mean and variance are equal, while quasi-likelihood estimation provides consistent parameter estimates and adjusted standard errors in overdispersion cases where data violate the mean equal to variance assumption. Over-dispersion is common in clustered data and also frequently occurs in spatial data sets.

Poisson regression models were fit by quasi-likelihood estimation to test the univariable significance of a variety of predictors on the change in mortality rate of dairy cows on-farm during a heat wave compared to a control period. Pre- and post-heat wave periods were considered equally to account for possible survival effects (“survival of the fittest”), and to improve the predictive ability of the model compared to a “normal” period. These predictors included: locations expressed as easting and as northing, predicted 3-day average HSI, year, and a yes/ no binary variable that was an indicator for the presence of a heat wave. Variables that appeared significant at a relaxed significance level of α = 0.2, were entered into a multivariable Poisson regression model. The Poisson regression model was fit using backward stepwise modeling procedures until all remaining variables in the model were significant (α = 0.05). A random effect was used for farm. Farm herd size was accounted for in all models as the natural log of the number of cows. Fit of the model was assessed using a quantile-quantile plot of residuals.

All analyses were done in R Statistical Package [22], using a significance value of α = 0.05, unless otherwise stated.

To test for geographical areas of high risk, a spatial scan test [23] was applied across Southern Ontario to determine if certain areas were at increased risk of heat stress related mortality. The mortality ratio was estimated by the spatial scan test based on the number of mortality records per farm and the number of cows on that farm aggregated over the 3 heat waves. Spatial coordinates in latitude and longitude were translated to Cartesian coordinates in Universal Transverse Mercator (UTM) 17 North, for analysis. A purely spatial scan test (i.e. no temporal analysis) using the Poisson distribution was used to test for clusters of high or low incidence of on-farm mortality. A temporal scan test was not performed. Analysis was done using 999 Monte Carlo experiments and a maximum scanning window of 50 % of the population. Analysis was conducted in SaTScan version 9.3 [24] using a significance value of α = 0.05.

## Results

### Descriptive results

**Fig. 2:**
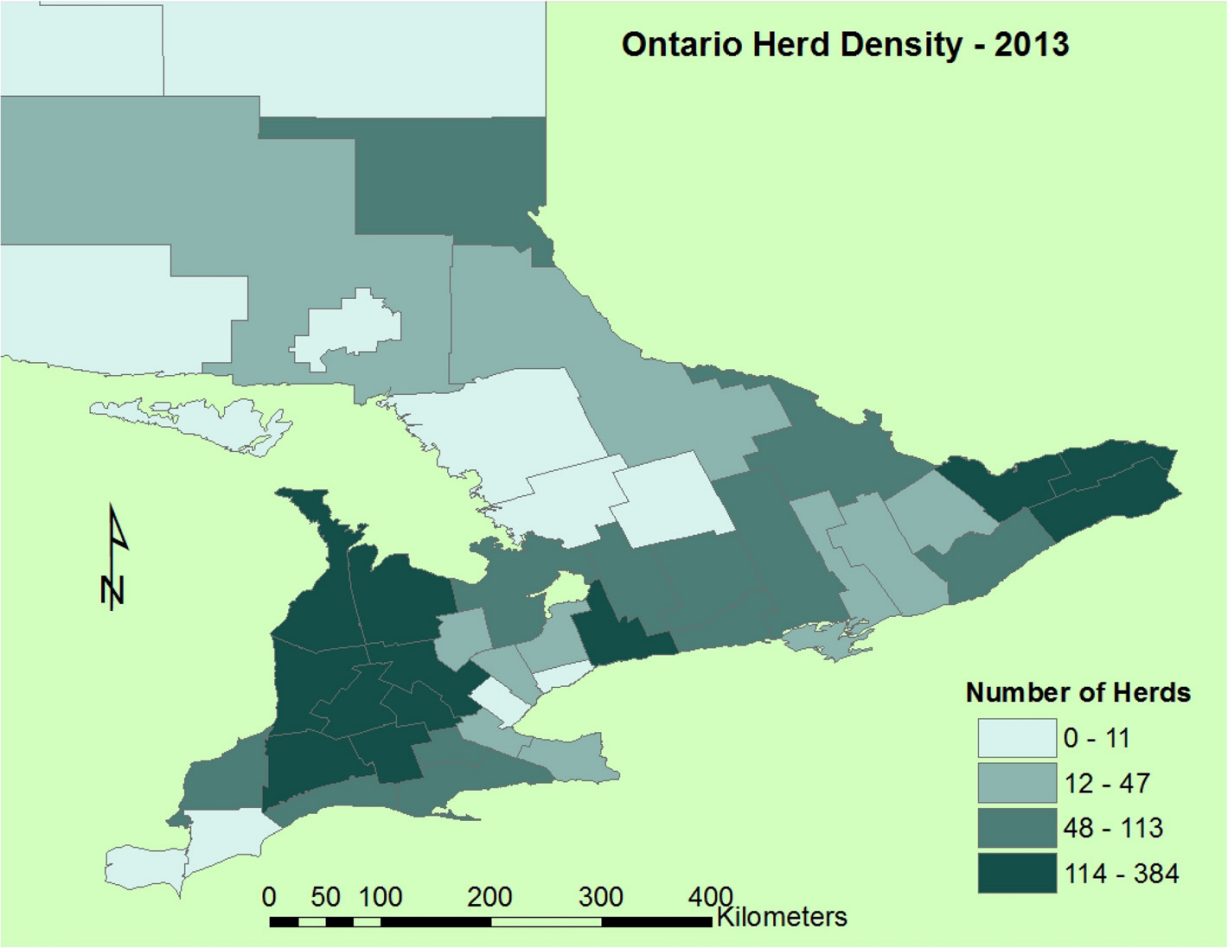
Dairy cow farm density map with total number of dairy farms per county

**Fig. 3:**
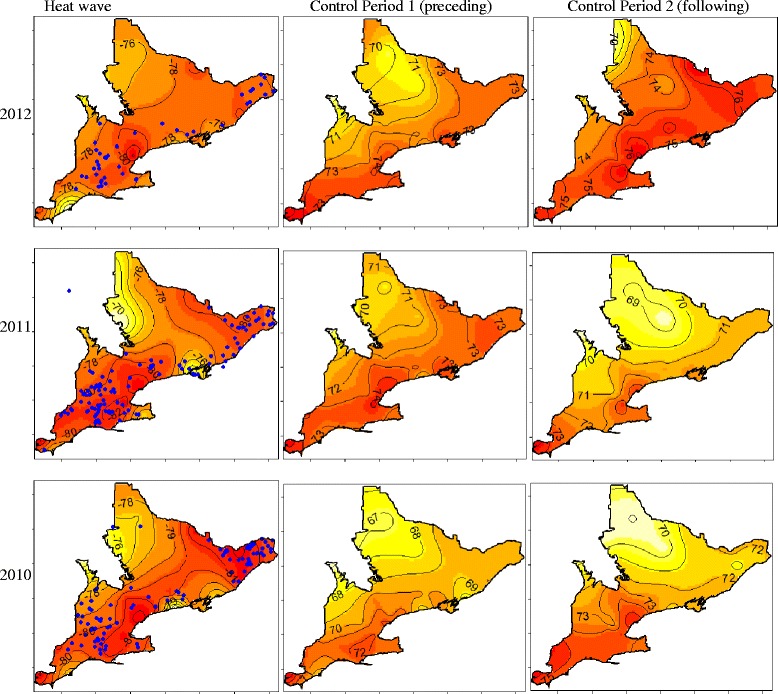
Heat stress maps for Southern Ontario during each of the 3 heat waves and 6 control periods with farm locations reporting deaths in that period marked in blue

**Fig. 4:**
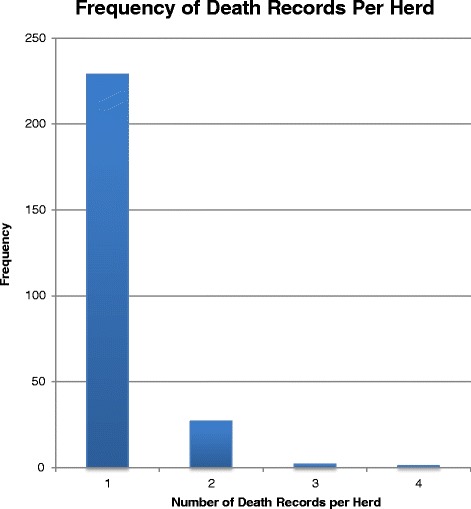
Distribution of dairy cow death records per herd during a heat wave

**Fig. 5:**
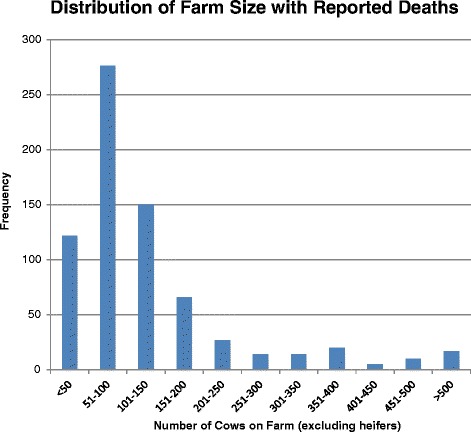
Farm size for herds with reported deaths (excludes heifers)

A total of 281 death records for three heat waves from 2010 to 2012 (Table 2) were retrieved from the DHI database. In total, 47 deaths were recorded on 2,953 farms on the 9 dates in 2012, 126 deaths on 2,894 farms in 2011 and 108 deaths on 2,842 farms in 2010. During a heat wave the average total number of on-farm dairy cow deaths registered with DHI across Southern Ontario was 94 deaths per 3-day period (Table 2). The average is thus approximately 1 death per 30 farms. Deaths during all heat waves followed a similar spatial pattern to the density of dairy farms (Fig. 2) and the distribution of heat stress in Southern Ontario during a heat wave [19]. As a result, on-farm dairy cow deaths most frequently occurred in the southwestern portion of Ontario or the central and northeastern areas (Fig. 3). Most cows were 3 years old at the time of death (i.e. mode) and the mean age of cows at the time of on-farm death was 5 years. Cows ranged in age from 1 year to 15 years at the time of death. Holstein-Friesian represented 94 % of on-farm dairy cow deaths, and similarly, 94 % of the dairy cow population in Southern Ontario is Holstein-Friesian [12]. Of all farms included in the study, 229 farms reported only 1 death over the 27 days of the entire study period. The maximum number of deaths on a single farm during a heat wave was 4 (Fig. 4). Farms included in this investigation ranged in size from 22 cows to 1,268 cows, while deaths were most frequently reported on-farms with 51–100 cows (38 %; Fig. 5).

**Table 2:** Number of farms reporting on-farm dairy cow deaths across Southern Ontario during each heat wave and the number of control farms for each year

Year	Heat wave	Number of controls
2012	47	94
2011	126	252
2010	108	216

As reported previously [19], heat stress in Southern Ontario follows a predictable pattern during heat waves. Heat stress is highest around major metropolitan areas such as Toronto and is also concentrated in the areas of southwestern Ontario near London and in the northeast around Ottawa. Maps illustrating heat stress distribution during each of the 9 periods and locations of on-farm dairy cow deaths in Southern Ontario are displayed in Fig. 3.

### Analytic results

In univariable Poisson regression analysis the variables HSI and Cartesian coordinates (i.e. northing and easting) were significant predictors of increased on-farm dairy cow mortality. As a fixed-effect, year was not statistically associated in univariable modelling with on-farm dairy cow mortality rates. Spatial coordinates suggest a trend in the mortality where easting from the most eastern point to the most western point of the study region predicts an increase in mortality rate of 8 % (1.08 times; *p* = 0.01; Table 3). When considering only the HSI in univariable quasi-likelihood Poisson regression, a one unit increase predicts a 3 % (1.03 times; *p* ≤ 0.001) increase in on-farm mortality rate. Backwards elimination using the full model with (with HSI, easting and northing predictors) left the HSI predictor as the only significant variable (*p* ≤ 0.001), see Table 4. A spatial Poisson regression model was attempted, but it did not fit the data well and was rejected for substantial underdispersion.

**Table 3:** Univariable analysis of on-farm mortality rate (Non-spatial poisson); Final model

Parameter	Coefficient	IRR	95 % CI	*p*-value
HSI	0.0287	1.03	1.025–1.035	<0.001
Cartesian Coordinates	X = 0.08070	X = 1.084	X = 0.019-0.147	X = 0.0102
	Y = −0.06658	Y = 0.9356	Y = −0.236-0.102	Y = 0.4123

**Table 4:** Multivariable analysis of on-farm mortality rate (Quasi-likelihood poisson)

Parameter	Coefficient	IRR	95 % CI	*p*-value
HSI	0.3878	1.47	1.417–1.523	<0.001
(X,Y) Cartesian Coordinates	X = 0.04249	X = 1.04	X = 0.980-1.010	X = 0.149
	Y = 0.06748	Y = 1.07	Y = 0.908-1.232	Y = 0.387

In the final model, a one unit increase in HSI results in a 3 % increase in the on-farm mortality rate (CI_95%_ (IRR) = (1.025,1.035); *p* ≤ 0.001), see Table 3C. The average HSI on-farm during a control period was 71.1 units and during a heat wave 79.7 units. This average 8.6 units difference (79.7–71.1 = 8.6) in HSI between a control period and a heat wave suggests an increased on-farm dairy cow mortality rate of 27 % during heat waves.

No significant circular clusters (*p* = 0.439) were located in Southern Ontario for on-farm dairy cow mortality during heat waves.

## Discussion

Dairy cow mortality is associated with heat wave related heat stress and follows a predictable pattern. While a single unit increase in HSI may have limited impacts on the number of farms affected by on-farm mortality, a substantial increase such as the average 8.6 unit increase in HSI accompanying a heat wave has dramatic impacts on farmers. From a population-level perspective, a 27 % increase in mortality during a heat wave will on average result in an additional 26 deaths in southern Ontario per heat wave. Since most deaths were one per farm, this suggests that approximately 26 additional farms will be affected. That is approximately 1 % of all farms affected by an excess death per heat wave. In contrast, all farms in Southern Ontario will be affected by milk production losses during a heat wave. This increased impact is part of the loss of productivity associated with heat waves as it does not include such negative effects as decreased milk productivity, reduced fertility and additional morbidity.

Similarly, while the HSI is a better predictor for on-farm dairy cow mortality than Cartesian coordinates, farms located further west are at increased risk of mortality. Both heat wave status and Cartesian coordinates are redundant to the spatially varying his, which was predicted to farm locations using geostatistical kriging of weather station data. The heat wave indicator is a binary variable and thus carries less information than the his, which is a continuous variable that more precisely measures heat related discomfort. Cartesian coordinates only allow modelling of a linear trend surface related to spatial variation of mortality. On the other hand, the HSI is spatially varying, but its geographic pattern is more complex than an artificial linear trend surface. Therefore, HSI is a better descriptor for mortality than the heat wave binary variable or the spatial coordinates. For the same reason, a spatially correlated Poisson regression model was not adequate for modeling on-farm dairy cow mortality: as the HSI accounts for spatial variation in the model, additional spatially correlated random effects would result in an over-parameterized model. Indeed the range of the spatial Poisson regression model was negligible, i.e. shorter estimated than minimum inter-farm distances.

Year was not statistically associated with on-farm dairy cow mortality in univariable regression models (and was not tested as a random-effect either). A non-significant fixed effect for year likely indicates that there were negligible changes in the Ontario dairy herds between 2010 and 2012. Changes such as herd size, barn structure, and dairy population should have been accounted for in this way. This is reasonable since there are over 4000 dairy farms in Ontario, and the study period was only 3 years in length. By dropping the non-significant predictor, minimal changes were seen in the coefficients of remaining predictors. A random effect for year was not modelled as interest here is to demonstrate the association between heat wave occurrence and related increases in on-farm dairy cow mortality during 2010–2012. However, since control periods were specific to the heat waves the results should also indicate the anticipated excess mortality for near future heat waves.

No significant circular clusters were found for increased or decreased mortality in Southern Ontario for on-farm dairy cow mortality. The reason no significant clusters were found may have to do with the circular restrictions of the scan test applied in SaTScan [24]. The application of an irregularly shaped scan test such as the elliptic scan test is an alternative method; however there is no justification for testing an irregularly shaped cluster window of weather phenomena. A single scan test was applied to the data because of the similarity of spatial heat stress patterns in Southern Ontario over time [19]. A large number of deaths occur in the southwest and northeast of Southern Ontario, areas where there are many dairy cow farms. An explanation may be that the entire study area seems to be affected by the necessarily broad scale weather systems which generate a heat wave. Therefore, small or localized clusters related to dramatic increases or reductions in temperature over 3 days are unlikely and thus, so are changes in the pattern of the outcome, mortality. While no spatial mortality clusters were detected, this does not diminish the relevance of a temporal relationship in which mortality of on-farm dairy cows increases with increasing HSI during a heat wave.

Holstein-Friesian cows are known to be the most susceptible dairy cow breed to heat stress [25]; however, no more deaths were observed in Holstein-Friesians than other dairy cow breeds in this study. In Ontario, other breeds are relatively rare and there may be insufficient power to test this.

This study provides evidence for the current impact of heat stress on dairy cow mortality in Southern Ontario. The finding of increased mortality with increasing HSI illustrates the potential need for more effective heat abatement strategies even under the temperate weather conditions of southern Ontario. This study most likely underestimates the severity of this problem as heat waves have been shown to cause further production losses on farm necessitating heat abatement strategies [5]. While it can be suspected that a variety of heat abatement strategies are applied in Ontario, their use pattern and effectiveness has not been systematically investigated. Heat abatement strategies have been shown to decrease productivity losses on-farm by 25 % when used intensively rather than minimally [7]. Other effective strategies in addition to traditional heat abatement strategies include management style, nutritional changes and improved breeding techniques [26]. If, in fact, heat abatement strategies are being employed and are reducing the number of deaths, than the impact of heat waves or heat stress on mortality may be underestimated. There is evidence that the HSI inside the barn is as much as 11 units higher than outside the barn [27]. This underestimation of the HSI should be constant across Southern Ontario, thus these estimates of the relationship between HSI and mortality from the Poisson regression modelling should not be affected. If regional impacts exist, they are likely the result of farm management practices or barn style rather than the direct result of in-barn to exterior HSI differences. Future work exploring farm management practices, barn style and environmental factors should be considered. It would also be interesting to investigate heat abatement strategies with respect to effectiveness, efficiency and sustainability.

This study makes a very conservative estimate of the on-farm mortality rates associated with heat waves in Southern Ontario. Research is suggestive that heat waves will increase in frequency, intensity, duration and size [15], all of which can increase the on-farm mortality rate beyond what is currently reported. This data set, i.e. the CanWest DHI, provides data on only 70 % of farms in Ontario [14], and thus probably estimates 70 % of the true on-farm mortality rate. The data does not include calves and heifers, which have also been reported to have increased mortality rates during extreme heat [8]. This is a welfare issue which merits further investigation and attention for dairy cows and other livestock species in Southern Ontario.

In light of the relative increase in mortality associated with current heat stress levels, predictions of future increases in heat waves and heat stress days highlight the importance of this issue for decades to come. Since 2003, there have been 1 or more heat waves per year in Southern Ontario, each threaten farm animal production and welfare in the province. This trend is predicted to increase substantially over the next number of years [15–18]. As heat stress increases, on-farm mortality rates are illustrative of a “tip of the iceberg” for other economic losses.

Heat wave warnings issued through a variety of media channels have been proven to decrease the risk of heat-related mortality [28]. It is reasonable to expect that the same would be true if these warnings would be coupled with a warning for farm animals.

This study comes with certain limitations. To estimate the heat stress, data from 37 weather stations was available. It would be advantageous to have more weather stations to reduce spatial interpolation errors in heat stress values. However, it would be even better to adjust for the exact in-barn heat stress rather than working with the ambient or outdoor heat stress. Such data is, however, currently not available. The same holds for basic epidemiological risk factors such as age, sex and breed, which are generally used to derive standardized mortality rates. This study investigates dairy cows and they are by nature all female and almost exclusively of the Holstein breed (94 %) in Ontario. Age (in years or by lactation) was not considered since it was not believed that the age distribution at population level in the Ontario industry would change over the short study period of 3 years. Including age as a covariate in future studies is recommended.

## Conclusion

Heat wave induced heat stress is an existing problem which poses underestimated threats to the dairy industry in Southern Ontario. During 3 consecutive heat waves 2010–2012 in Southern Ontario, a one unit increase in HSI was estimated to increase the mortality rate by 3 % (1.03 times) and the typical rise associated with a heat wave increased the mortality rate by 27 % compared to a control period. On-farm mortality rate increases by 8 % (1.08 times) for easting across the region (approximately 400 km).

While climate change models unanimously predict an increase in the frequency and severity of extreme weather events such as heat waves, this problem will continue to grow in Southern Ontario. In consequence the excess mortality rate due to heat stress increases, so too do the combined economic losses associated with heat stress in farm animals. While farmers may employ heat abatement strategies, the effectiveness of these measures is unknown in Canada. Moreover, if these measures are in place during a heat wave, the excess mortality due to heat stress in Southern Ontario may be underestimated and differential use of these measures could impact the apparent spatial patterns.

## Abbreviations

HSI: Heat stress indicator
DHI: Dairy herd improvement
IRR: Incidence rate ratio
THI: Temperature humidity index
GPS: Global positioning system
UTM: Universal transverse mercator
